# Precision medicine and therapies of the future

**DOI:** 10.1111/epi.16539

**Published:** 2020-07-24

**Authors:** Sanjay M. Sisodiya

**Affiliations:** ^1^ Department of Clinical and Experimental Epilepsy UCL Queen Square Institute of Neurology London UK; ^2^ Chalfont Centre for Epilepsy Bucks UK

**Keywords:** anti‐seizure drugs, failure, genetics, personalized, pharmacogenetics, surgery

## Abstract

Precision medicine in the epilepsies has gathered much attention, especially with gene discovery pushing forward new understanding of disease biology. Several targeted treatments are emerging, some with considerable sophistication and individual‐level tailoring. There have been rare achievements in improving short‐term outcomes in a few very select patients with epilepsy. The prospects for further targeted, repurposed, or novel treatments seem promising. Along with much‐needed success, difficulties are also arising. Precision treatments do not always work, and sometimes are inaccessible or do not yet exist. Failures of precision medicine may not find their way to broader scrutiny. Precision medicine is not a new concept: It has been boosted by genetics and is often focused on genetically determined epilepsies, typically considered to be driven in an individual by a single genetic variant. Often the mechanisms generating the full clinical phenotype from such a perceived single cause are incompletely understood. The impact of additional genetic variation and other factors that might influence the clinical presentation represent complexities that are not usually considered. Precision success and precision failure are usually equally incompletely explained. There is a need for more comprehensive evaluation and a more rigorous framework, bringing together information that is both necessary and sufficient to explain clinical presentation and clinical responses to precision treatment in a precision approach that considers the full picture not only of the effects of a single variant, but also of its genomic and other measurable environment, within the context of the whole person. As we may be on the brink of a treatment revolution, progress must be considered and reasoned: One possible framework is proposed for the evaluation of precision treatments.

**Key Points**

- Precision medicine, empowered by genetics, has produced important benefits, and holds much promise for future treatment advances.
- Precision medicine approaches are not always available, and not always successful: Failures and problems need to be evaluated and reported.
- Complexities in precision medicine need to be acknowledged and addressed looking beyond single genetic variants and presumed pathophysiology.
- A framework is needed for precision medicine, with proof that treatment is actually addressing the presumed epileptogenic pathophysiology.

## 1 INTRODUCTION

**Figure 1:**
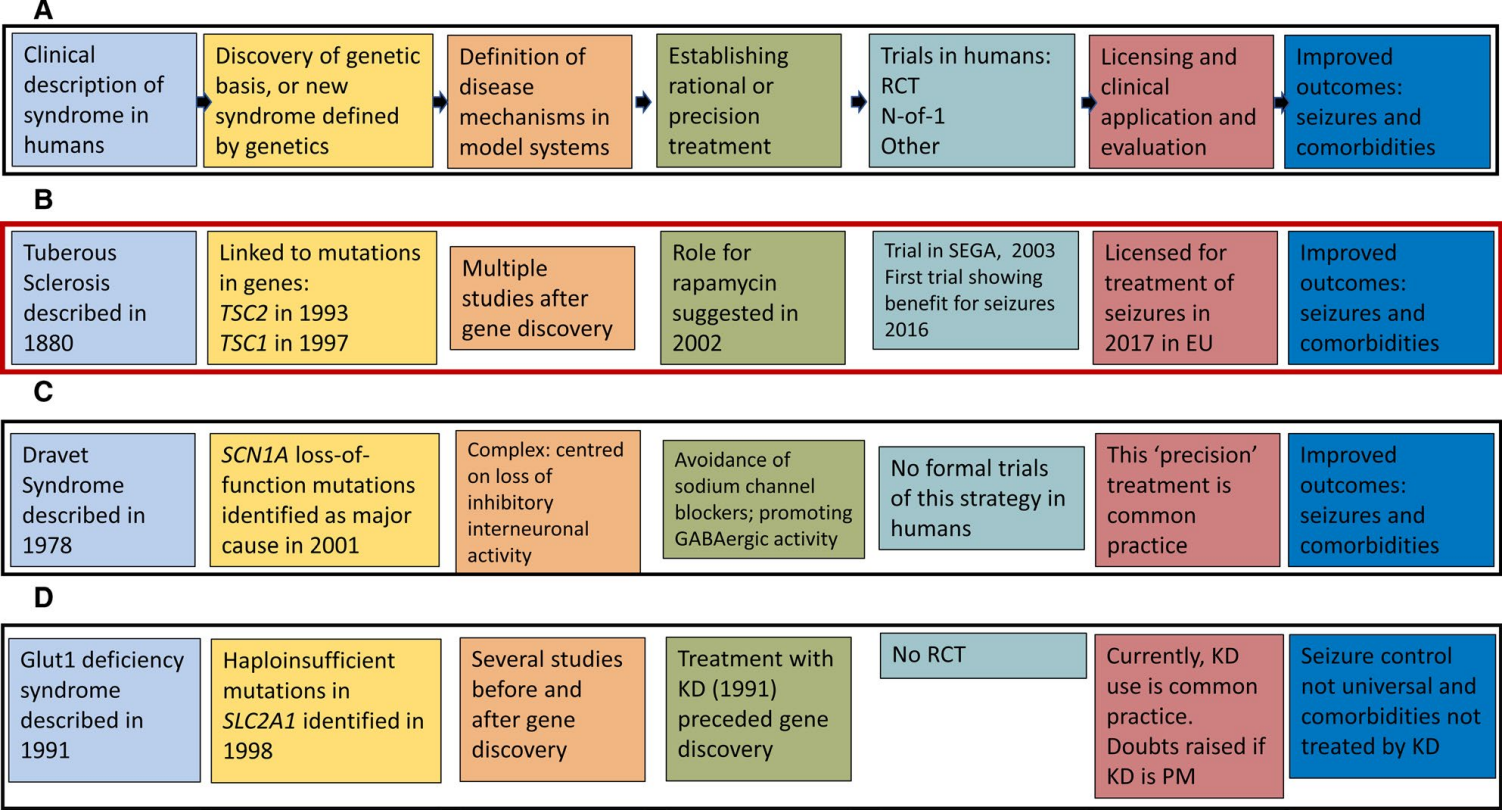
Typical current PM scenarios. The same color scheme is used throughout for each of the steps crystalized in this figure. A, The idealized paradigm, with a linear progression from clinical description of an epilepsy, to determination of its genetic cause, a definition of necessary and sufficient disease mechanisms, establishing the basis of a rational treatment, subsequent clinical trials, licensing, and seizure‐free outcomes with improvements of comorbidities. B, Realization of the paradigm in tuberous sclerosis. It is worth noting the actual timeline, which is not depicted linearly. Also noteworthy is that although everolimus is licensed for use for seizures in tuberous sclerosis, the necessary funding mechanisms are not always in place. C, Application of the linear paradigm in Dravet syndrome due to mutations in *SCN1A*. The pathophysiology of Dravet syndrome has been shown to be more complex than originally reported, but the reasoned strategy of avoidance of sodium channel–blocking antiseizure drugs is still typical practice, with published, although not from formal trials, evidence of benefit.^36^, ^68^ Agents for which randomized controlled trials have been undertaken in Dravet syndrome, such as stiripentol, cannabidiol, and fenfluramine, are not PM within the draft framework outlined at the end of this document. D, The paradigm in GLUT1 deficiency syndrome. Ketogenic dietary therapy (KD) was instituted in people with the clinical syndrome on biochemical grounds before discovery of the genetic cause. KD is considered standard treatment for GLUT1 deficiency disorder, although there have been no randomized controlled trials. Its frequently quoted position as a PM has been reconsidered.^35^ Abbreviations: EU, European Union; KD, ketogenic diet; PM precision medicine; RCT, randomized‐controlled trial; SEGA, subependymal giant cell astrocytoma

Precision medicine (PM) has been defined as an approach that uses a person's genetics, environment, and lifestyle to help determine the best approach to prevent or treat disease.^1^ There have been well‐documented,^2^ and occasionally remarkable,^3^ examples of the success of PM in the epilepsies. Dramatic progress in the field has been empowered by remarkable achievements in epilepsy genetics.^4^ The application of genetics has identified the cause of many severe, often treatment‐resistant, human epilepsies, and generated a paradigm for PM stretching from genetic discovery, to in vitro and in vivo models, through rational drug selection, repurposing, or discovery, to clinical trials or PM use in people with epilepsies due to mutations in the gene in question (Figure 1 and http://epilepsygenetics.net/2014/06/11/precision‐medicine‐in‐genetic‐epilepsies‐three‐criteria‐to‐consider/#more‐3125). In other instances, genetic discovery has explained why some therapies may be more effective than others, or has demonstrated how a treatment used before identification of the cause of a disease may be typically rebranded as a PM.^5^ The potential of PM seems huge. Enthusiasm for PM is apparent and understandable: We would all like to provide rational effective care for each individual person with epilepsy.

The aim of PM is treatment of epilepsies in humans, rather than PM solutions to engineered or spontaneous epilepsies in model systems. Across human disease, PM has typically been driven by discovery and progress in genetics as the contemporary enabling instrument. But whether we consider the field today, or whatever form it will take in the future as new areas of understanding are brought into management strategies, PM, as best clinical practice, has always been to offer individualized treatment, employing the best available tools and understanding of disease biology: As Sir William Gowers said: “Strive by every method you can think of to gain the utmost certainty attainable…..whether the diagnosis of a disease or the action of a drug; or at least, relentlessly expose, and candidly admit to yourselves, the degree of uncertainty.” PM is not a new idea—it is a new label for a longstanding idea, given a tremendous boost and direction by genetics. But, echoing Gowers, although there have been some most welcome advances in treatment of some epilepsies, the whole picture is more complex, and there are lessons to be learned from failures of the current approach to PM.

PM as now conceived is transformational. Such paradigm shifts in scientific thinking typically require the wider community of stakeholders to revise their own thinking.^6^ Unless this happens, the full potential of novel ideas can remain unrealized. Equally, investing PM with unrealistic expectations risks generating disappointment and disengagement. Progress in science and its dissemination are subject to trend and popular excitement as is any other field of human endeavour. We may expect our research to be grounded in fact, but there is a crisis of reproducibility across science.^7^ The selection of areas receiving editorial attention is also to an extent arbitrary, whereas many other factors influence publication, including its economics.^8^ The rise and fall of interest in previous apparent revolutions in epilepsy bears witness to the idea that a particular excitement may be only a passing fashion.^9^ PM perhaps gets closer to disease biology than any other previous movement in the epilepsies, but if it is truly to change the way we think about epilepsy, clarity is needed about what PM is and how it should be developed: Some thoughts are presented here.

## 2 PRECISION MEDICINE AND THE PIVOT OF GENETICS

**Figure 2:**
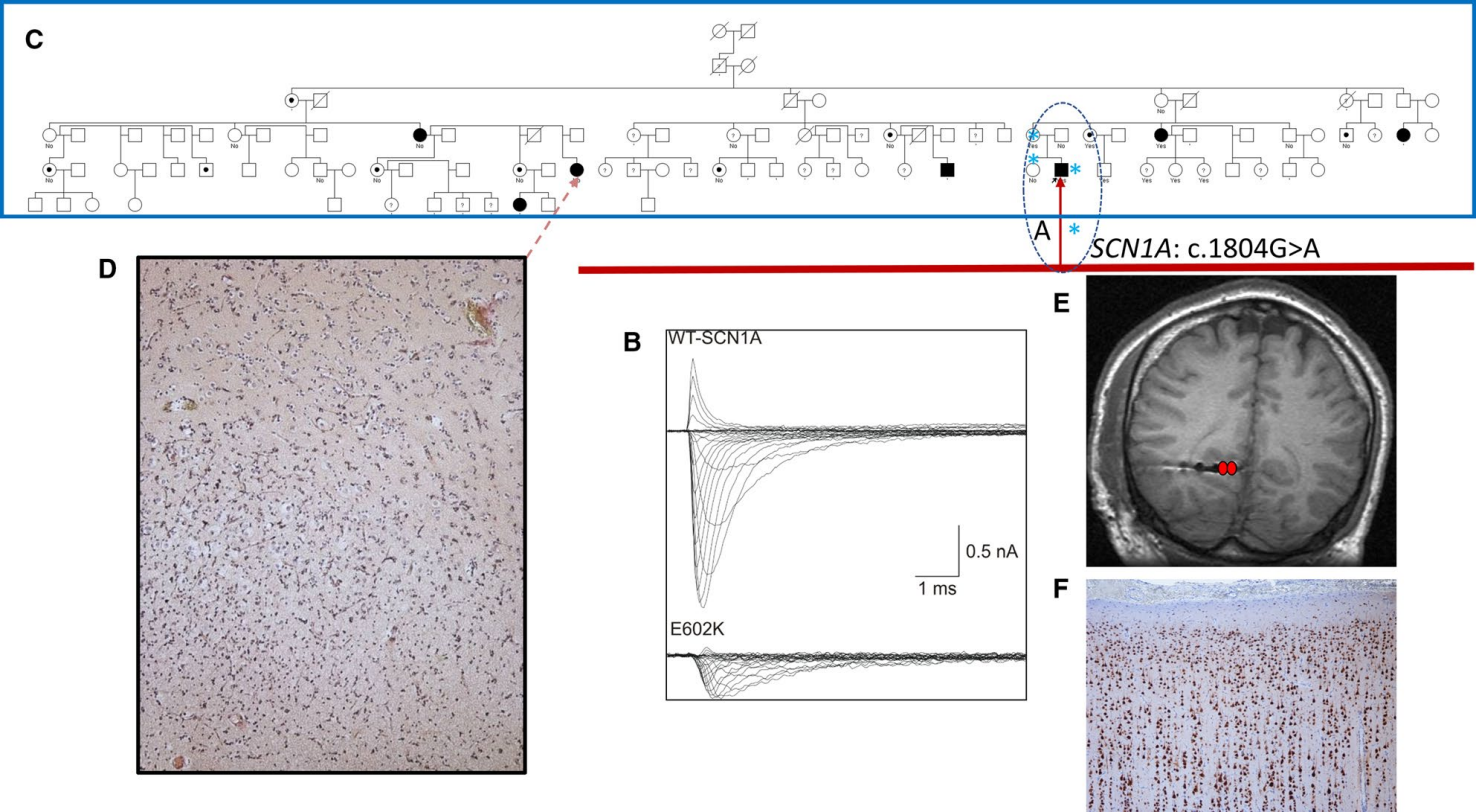
An example of complexity and potentially misleading genetic information. The proband had severe treatment‐resistant right occipital lobe epilepsy, referred on a combination of five antiseizure drugs, including three sodium channel–blocking drugs. His epilepsy was life‐threatening, with recurrent episodes of generalized tonic‐clonic status epilepticus, including an episode with acute renal failure. High‐resolution brain magnetic resonance imaging (MRI) was unremarkable. He had a personal and family history of febrile seizures in his sister and mother. At the time of referral, only single gene testing was available; gene panel, exome, and genome sequence were available, and neither ExAC nor gnomAD were in existence. In view of the history in the proband and nuclear family, *SCN1A* was Sanger sequenced in the nuclear family. The heterozygous missense mutation (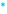 in A, dotted oval) c.1804G>A, p.Glu602Lys was identified in the proband, his mother, and sister, and not in the asymptomatic father. Current evaluation shows that the variant absent from gnomAD, has a CADD score of 13, and a REVEL (https://sites.google.com/site/revelgenomics/) score of 0.399: 75.4% of disease‐causing variants, and only 10.9% of neutral variants have a REVEL score of >0.5; variants proven to have a functional consequence, as the gold standard measure of pathogenicity had CADD scores >20 and REVEL scores >0.7 in a recent study of clinical prediction of the effect of variants in *SCN1A*.^36^ On bioinformatic grounds, this variant would be considered unlikely to be deleterious. These measures were unavailable at the time the proband was seen. Functional testing of the variant in vitro showed loss‐of‐function (B; Ref.^69^). At this point, the full pedigree (C, blue rectangle) became available through the proband's maternal grandmother. The *SCN1A* variant found in the proband was absent in the maternal grandmother, from whom a family history of right occipital lobe epilepsy (filled symbols) or migraine, inconsistently lateralized, without features of seizures (dotted symbols) was obtained. It emerged that a distant relative (dotted arrow) had had a right occipital lobectomy elsewhere with full control of generalized tonic‐clonic seizures, and the identification of a dysembryoplastic neuroepithelial tumor on histological examination (D). Pre‐surgical evaluation was then undertaken in the proband, with confirmation from intracranial electroencephalography (EEG) (E) of mesial right occipital lobe seizure onset: The electrode track is visible—seizure onset was from contacts colored red. Having regularly experienced ictal and postictal hemianopia, and with the knowledge of benefit from surgery in the distant relative, the patient selected an occipital lobectomy, and has been free of all seizures for over 10 years, except during one episode of diarrhea. He has a fixed right homonymous hemianopia and remains on the five antiseizure drugs on which he was referred, not wishing to come off any. Histology of the resection specimen was unremarkable: There was no evidence of a dysembryoplastic neuroepithelial tumour (F). Although the *SCN1A* variant identified causes loss‐of‐function in vitro, it is not the cause of the familial epilepsy shared by the proband; it may be responsible for the nuclear familial febrile seizures, but had only that information been acted upon (eg, with withdrawal of the sodium channel–blocking antiseizure drugs), it seems unlikely that he would have become seizure‐free, as he had a cluster of generalized tonic‐clonic seizures during the episode of diarrhea some years after surgery. The genetic cause of the epilepsy in the family has not yet been identified, despite exome sequencing of several family members

As the putative genetic causes of more and more, especially rare, epilepsies have been identified, hope has grown that additional rational therapies will emerge.^10^ Genetic discovery sets the direction for selected or engineered solutions. Thus if a particular epilepsy is shown to be due to pathogenic variants in a gene encoding an ion channel, for example, the well‐trodden pathway is to determine whether a particular phenotype is due to loss‐ or gain‐of‐function of the encoded mutant protein, and then to identify a treatment that can reverse this proximal functional consequence.^11^ Such a treatment may be an antiseizure drug already known to have the desired effect on the channel in question,^12^ or a repurposed drug (such as 4‐aminopyridine use in epilepsy due to gain‐of‐function mutations in *KCNA2* ^13^); or it may be that a new treatment needs invention. These strategies can take considerable time and effort to accomplish, with notable complexity possible even for a single mutation in one gene.^14^, ^15^ Moreover, finding a mutation does not necessarily mean it is the cause of the patient's epilepsy, even with in vitro evidence that there is a change in channel physiology: Additional evidence may be required, such as segregation of the variant within a family (Figure 2). If the pathogenic variant is in a gene encoding part of a metabolic or vitamin‐related pathway, for example, then there may be apparently simple treatments that can replace what is missing or bypass the relevant step(s) in the pathway (for example, in epilepsies due to mutations in the gene *GAMT*). In other instances, there may not yet be an obvious immediate treatment option (for example, epilepsies due to mutations in the genes *PURA*, *CHD2*, and many others).

Journals publish gene discoveries regularly. They also prefer to report success: Publication bias is well‐studied and longstanding, especially in clinical trials.^16^, ^17^ For researchers, there is also pressure to publish, a phenomenon across science.^18^ These pressures have an impact on the development and popularization of PM. A search under “precision medicine epilepsy review” in PubMed on 30th January 2020 generated 65 references on a conservative evaluation, a number far exceeding the actual number of PM available currently in the epilepsies. Most such reviews focus on the role of genetics (the author accepts responsibility for two of these).

Reports of gene discoveries, and of PM approaches that work and bring about sometimes dramatic outcomes, are naturally engaging, usually high‐profile, and drive the field forward. Failure of PM is less often reported, but may feature in a second wave of negative or cautionary reports following early data and initial clinical promise, with increasing watchfulness expressed by many succeeding studies (see Table 1 for the use of quinidine in epilepsies due to gain‐of‐function mutations in *KCNT1* as an example). Initial success and bias against publication of negative outcomes, can lead to a perception that PM is both often feasible and truly precise, constituting incisive and much‐desired “magic bullet(s).” One view of PM, arising through successes driven by genetic discovery, is that we simply need to be able to bring together the right group of experts with sufficient funding and motivation to pass in linear fashion from genetic discovery to precise treatment (Figure 1). This can work, but the situation is often more nuanced. Overall, these concerns make PM a more complex undertaking than may currently seem the case, and take us beyond genetics, or at least a single variant, alone. It is worth remembering also that genetics has often focused on tractable syndromes and favorite phenotypes; until more recently, it has rarely been applied to the less‐defined epilepsies, or systematically for everyone with epilepsy attending clinics, without some form of selection bias.

**Table 1:** Review of published cases with *KCNT1* gain‐of‐function mutations who underwent treatment with the putative PM quinidine

Reference year and number	Patients treated with Q	Mutation(s)	Response to quinidine (Q)	Overall support of quinidine treatment for *KCNT1* GoF mutation
Milligan et al 2014^76^	No patients studied—first report of effect of quinidine	c.2688G>A (M896I), c.1193G>A (R398Q), c.2386T>C (Y796H), c.2782C>T (R928C), c.1283G>A (R428Q), c.2800G>A (A934T), c.2771C>T (P924L)	Significant block by 300 µmol/L Q of average current amplitude for all mutations tested	Proposal that Q might be a PM for epilepsies due to *KCNT1* GoF mutation
Bearden et al 2014^77^	One patient	c.1283G>A; R428Q	Seizure‐free for 4 mo	“Dramatic improvement with quinidine, the patient did not initially achieve complete seizure freedom, and her development has remained severely delayed”
Rizzo et al 2016^78^	No patients treated with quinidine, but two variants studied in vitro	c.862G>A; G288S c.1546A>G; M516V	Reduction in channel currents	"result suggests two genotype‐tailored pharmacological strategies to specifically counteract the dysfunction of KCNT1 activating mutations in MMPSI patients"
Mikati et al 2015^79^	Two patients	c.2386T>C; Y796H	No benefit	“Although the current limited data do not appear to support the idea of substantial clinical benefit of quinidine, quinidine does clearly illustrate a new potential paradigm for the development and clinical evaluation of genetically targeted therapies in epilepsy”
		c.1887G>C; K629N	80% reduction in seizure frequency	
Chong et al 2016^80^	One patient	c.1283G>A; R428Q	No benefit	Suggested that earlier initiation with Q may be needed for effect
Fukuoka et al 2017^81^	One patient	c.1955G>T; G652V	>50% reduction in ‘epileptic spasms’, but not seizure‐free	..Quinidine “therapy should be attempted in patients with West syndrome caused by KCNT1 mutations..”
Baumer et al 2017^82^	One patient	c.2278A>T; I760F	No benefit	“Given minimal improvement in seizures and development, quinidine was stopped.”
				Bluish discoloration of hands, feet, lips
Madaan et al 2018^83^	One patient	c.808C>G; Q270E	No benefit	“Neither quinidine nor ketogenic diet could control his seizures”
Mullen et al 2018^84^	Six patients	Five patients from one family: c.2782C>T; R928C c.2849G>A; R950Q	No benefit	“Quinidine did not show efficacy in adults and teenagers with autosomal sominant nocturnal frontal lobe epilepsy (ADNFLE). …. Although small, this trial suggests use of quinidine in ADNFLE is likely to be ineffective coupled with considerable cardiac risks.”
McTague et al 2018^85^	Three patients (of 12 with *KCNT1* mutation)	c.820C>A; L274I	No benefit	
		c.1504T>G; F502V	“Marked reduction in seizures”	
		c.2687T>A; M896K	No benefit	Marked in vitro blockade, but clinical response only initially on Q treatment
Abdelnour et al 2018^86^	Three patients	c.1421G>A; R474H	No benefit	“The above‐mentioned findings support performance of prospective controlled studies of quinidine efficacy in children with KCNT1 gain‐of‐function mutations that control for age as a possible variable affecting response”
		c.2955G>T	Seizure frequency reduced by >50%, and less severe; duration of follow‐up not stated	
		c.1193G>A; R398Q	No benefit	
Ko et al 2018^87^	Two patients treated with Q	c.1421G>A; R474H c.2800G>A; A934T	QT prolongation before target dose reached	“Unfortunately, quinidine was not effective in 2 patients with migrating focal epilepsy in infancy related to *KCNT1* mutations”
		c.1038C>G; F346L (not stated which two patients treated with Q)	No benefit	
Dilena et al 2018^88^	Two patients	c.2849G>A; R950Q	Complex treatment course; 90% reduction at best	“Multicenter studies are needed to establish a consensus protocol for patient recruitment, quinidine treatment modalities, and outcome evaluation, to optimize clinical efficacy and reduce risks as well as variability associated to quinidine use in such severe developmental encephalopathy”
		c.2677G>A; E893K	>85% reduction	
Numis et al 2018^89^	Four patients	c.1649‐1651delAGC: Del550	“No significant changes in seizure frequency and severity during therapy”	“Despite early targeting of the pathologic channel, no disease‐modifying effects were noted in the clinical phenotype. In contrast to previous hypotheses, our data do not support ‘age at quinidine initiation’ as a variable that can modify overall outcome”
		c.776 C>A: A259D		
		c.1546 A>G: M516V		
		c.1283 G>A; R428Q		
Yoshitomi et al 2019^90^	Four patients	c.1283G>A; R428Q	>60% reduction in seizure frequency	Q “…as a promising treatment option for some patients with EIMFS and West syndrome, however, quinidine may not be beneficial for patients with other focal epilepsies.”
		c.2800G>A; A934T	48.1% reduction in seizure frequency	
		c.862G>A; G288S	12% increase in seizure frequency	
		c.1420C>T; R474C	23.1% reduction in seizure frequency	
Jia et al 2019^91^	One patient	c.625C>T; R209C	Frequency of tonic seizures reduced by 75%	“…Therapeutic effect of quinidine may be influenced by multiple factors. The epilepsy phenotype, initiation age of therapy, and prior neuronal injury may all play a role in the efficacy of quinidine therapy. Henceforth, randomized controlled trials (RCT) should be performed to identify the relationship between the influencing factors and the efficacy of quinidine therapy”
Fitzgerald et al 2019^92^	Twenty patients	See their table 3 for full details—only seizure‐free responders listed here		“Until a precision therapy is developed with an improved side effect profile, thoughtful consideration of the potential benefits and risks of a trial of quinidine is reasonable in selected patients with *KCNT1*‐related epileptic encephalopathy. When initiating quinidine treatment in this population, we suggest a protocol‐based approach to maximize serum levels while monitoring for side effects”
		c.2795T>C; F932S [patient 5]	Only patient with seizure freedom > 3 mo	
		c.2881C>A; R961S [patient 6] c.2849G>A; R950Q [patient 12]	Initially seizure freedom > 1 mo, with a sustained response of >50% Initially seizure freedom > 1 mo, with a sustained response of >50% Two other patients had > 50% seizure reduction. All other patients had < 50% seizure reduction, no effect or worsened	
Datta et al 2019^93^	One patient	c.1283G>A; R428Q	Seizure exacerbation	“Timing of treatment and response may not be related” “alternative therapies to quinidine should be considered as a therapeutic option for patients with *KCNT1*‐related epilepsy”
Patil et al 2019^94^	Two patients	c.2849G>A; R950Q	80% reduction	“Large multicenter registries are needed to prospectively assess the impact of these novel therapies”
		c.2800G>A; A934T	30% reduction	
Passey et al 2019^95^	One patient	c.1066C>T; R356W [pathogenic] and c.2170_2184dup15; p.Pro724_Leu728dup [variant of unknown significance]	“Despite increasing doses and blood levels of quinidine over time, the longest period of seizure freedom for this patient has been about 3.5 d”	“…Minor relief with quinidine …important interaction with phenobarbital”
Kuchenbuch et al 2019^96^	Three patients (first two reported again in Barcia et al 2019)^38^	c.1283G>A; R428Q c.1420C>T; R474C c.1421G>A; R474H	No “improvement in seizures frequency or neurodevelopment”	“The poor prognosis of this developmental epileptic encephalopathy requires an urgent development of trials that should be used early at onset as the early control of seizures might improve the prognosis but should also be tested at later stages as epilepsy remains active in the chronic phase of this syndrome”
Summary	42 patients in total		14 patients experienced at least a 50% reduction in frequency of at least one type of seizure for a period of time (not always specified)	

## 3 COMPLEXITY

Trying a PM approach can sometimes prove more complex in reality than might have been anticipated. Efforts to understand why are rarely reported.

A single pathogenic variant in a single gene may initiate a disease process. Such a disease process in a person takes place in the context of the rest of that person's whole genome, with all its other variations and dynamicity of regulation and expression in both normal (eg, developmental) and response (eg, compensatory) programs.^19^, ^20^, ^21^ Moreover, by the time a disease is manifest and a molecular genetic diagnosis established, the pathogenic variant in question will have been active (or active and then quiescent) for some time. If the gene in question is involved in processes of development,^22^, ^23^, ^24^ then many fundamental organisational changes may have occurred^25^—as may have irreversible changes and some degree of functional compensation. Thus some rare severe epilepsies have been characterized as developmental, and not just epileptic encephalopathies,^26^ including some ion channelopathies,^27^ with complexity beyond initial conceptualization.^28^, ^29^, ^30^, ^31^ Discovery of a molecular genetic cause for a type of epilepsy, and determination of the molecular genetic cause of an individual's epilepsy, has been heralded as the end of the diagnostic odyssey.^32^ It is also the start of another, different, set of journeys and questions, which are likely to be complex, and for most of which we have not yet scoped out the landscape and may not yet have the tools to navigate^33^, ^34^: in this context, the GLUT1 deficiency syndrome, for which ketogenic dietary treatments are often considered precision medicine, has been thoughtfully reconsidered.^35^

In our current paradigm, the necessary first step after gene discovery is to secure functional evidence that the putatively pathogenic variants identified in the gene have a consequence. This proof can be as parsimonious as showing that the variants cause loss‐ or gain‐of‐function in vitro or in vivo, or alter gene expression, or cause a malformation. A valuable publication is likely to result, probably proving of high utility as others find that their patients carry variants in the same gene. A key difficulty is that in most subsequently reported cases, functional work is not performed, and pathogenicity is assumed. Although this approach may be valid in some instances, and, for example, in silico predictions may have been devised to serve as a proxy,^36^ the lack of functional information may be critical^37^—or it may be performed and be potentially misleading (Figure 2). Moreover, the reductionist approaches typically adopted in functional work cannot replicate the human condition in all its complexity. Thus, for example, the same mutation, sometimes with a proven functional consequence in vitro, may cause quite different clinical phenotypes, or sometimes no phenotype at all.^38^ One implication of PM must be that, we should, for example, seek out the mechanisms that lead a pathogenic variant segregating in a kindred to cause a mild epilepsy (eg, GEFS+, genetic epilepsy with febrile seizures+) in one individual, and a severe epilepsy (eg, Dravet syndrome) in a relative (*COL4A1‐*related disorder is another example).^39^, ^40^ Mosaicism may sometimes provide a credible explanatory mechanism.^41^ Additional influential genetic variants may also exist that modify a phenotype,^42^, ^43^, ^44^ or that cause what is in fact a digenic^45^, ^46^, ^47^, ^48^, ^49^ phenotype; presumably oligogenic phenotypes^50^ also exist in epilepsy. Human epilepsies, especially those that are severe and most in need of treatment breakthroughs, are typically associated with comorbidities. A reductionist approach does not (and usually cannot) explain these multiple strands of human “monogenic” disease. Circuit and animal models may also not capture this complexity—a significant proportion of human pathogenic variants, for example, have no consequences in mice.^51^

An assessment of success of precision medicine could include measures of its actual mechanistic effects. When PM is “successful,” there should be direct mechanistic evidence that it has achieved what it was intended to achieve. Seizures may cease, speech may emerge, gait may improve—but was this because treatment X reversed the dysfunction caused by putatively pathogenic variant Y in gene Z? Or because something else happened? Rarely is this proof obtained, or expected, in the people actually treated with the precision medicine. Equally, the PM approach may not be successful: We then need to provide a “precision failure” explanation. Failed PM attempts are published (eg, see Table 1 regarding *KCNT1*), often with considered speculation about the reasons for failure, but we do not know how many failed attempts are not published, and speculation may not transform into actual explanation. In the absence of a mechanistic explanation for these eventualities (or other outcomes not listed here), then can we truthfully claim the treatment is a precision therapy? Or are we in reality back to the situation where an existing drug brings about seizure control in an unexplained way in an ill‐understood epilepsy—for example, juvenile myoclonic epilepsy treated with valproate, which often works, although we do not know why, while we feel under no obligation to explain its failure. What can we honestly consider to be PM without the circular argument that it must be precise because it worked? What is necessary and sufficient?

## 4 PRECISION MEDICINE: BEYOND THE SINGLE VARIANT

**Figure 3:**
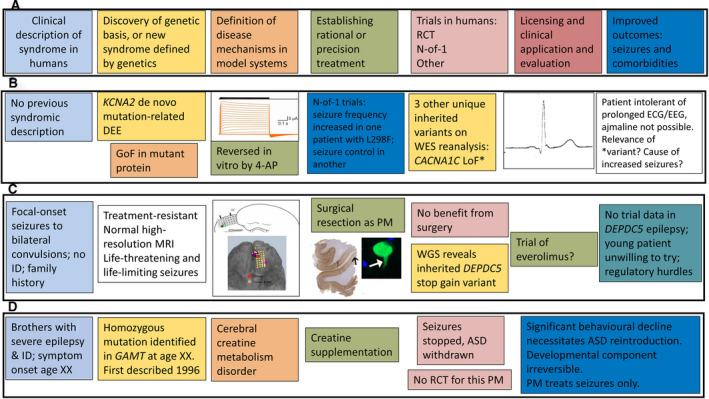
Real‐life examples of more complex PM scenarios. A, The linear PM development paradigm as detailed in Figure 1A. B, In a patient with frequent generalized tonic‐clonic seizures and a profound developmental and epileptic encephalopathy, with spastic quadriparesis, unable to walk and intolerant of most testing, with no syndromic diagnosis made, trio exome sequencing revealed a de novo *KCNA2* mutation (c.894G>T, p.Leu298Phe; Patient 6 in Ref.^70^) demonstrated to be gain‐of‐function in vitro, reversible by 4‐aminopyridine. Because of regulatory and other issues, time to provision of this putative PM for the patient was >18 months and was eventually funded by the family. However, the frequency of generalized tonic‐clonic seizures increased significantly on 4‐aminopyridine, which had to be discontinued. A child with the same mutation treated elsewhere with 4‐aminopyridine became seizure‐free (H. Lerche, personal communication). Review of the exome sequence data did not reveal any other de novo mutations, but identified three unique inherited variants, one of which (*CACNA1C* p.Gly490Arg inherited from an asymptomatic parent) has been shown previously to cause loss‐of‐function with a Brugada syndrome phenotype and shortened QT interval.^71^ The proband's electrocardiography (ECG) showed lateral early repolarization with prominent U waves, but no Brugada‐like changes, with a short QTc interval. However, further testing of the electroclinical significance of this variant in the patient has not actually proved possible. The cause of seizure aggravation by a putative PM effective in another patient with the same mutation has remained unknown. C, A young man with longstanding epilepsy with focal‐onset bilateral tonic‐clonic seizures had tried several antiseizure drugs. His mother was seizure‐free on monotherapy. As a PM strategy, he underwent surgical evaluation, including high‐resolution magnetic resonance imaging (MRI; unremarkable) and intracranial electroencephalography (EEG) recording that demonstrated focal frontal onset. Surgical resection led to a brief period of seizure freedom. Histopathology demonstrated an unusual pathology with early lipofuscin accumulation in dysplastic neurons.^72^ Postoperatively, whole genome sequencing revealed a *DEPDC5* stopgain mutation inherited from his mother. The different degrees of seizure control between mother and son are unexplained. Activation of the mammalian target of rapamycin (mTOR) pathway has already been demonstrated on tissue pathology in the proband.^72^ Given the life‐threatening nature of the proband's epilepsy, everolimus was considered. There is no trial or anecdotal basis for its use currently, no funding for its use, and no guidance for the duration of treatment that may be required, which were among the factors leading the patient to decline the offer to attempt to seek its individual use through clinical or research pathways. D, Two brothers were previously reported who were found to have homozygous mutation in *GAMT*. Seizure control was achieved with creatine supplementation.^73^ Antiseizure drugs were withdrawn without recurrence of seizures. Unmanageable behavioral deterioration necessitated reintroduction of some antiseizure drugs. In complex genetic epilepsies, it may be necessary to plan a PM approach in a multidisciplinary context, considering not only what might be done if the PM fails, but how life might change if PM succeeds and seizures are brought under control

Precision medicine should not consider as its narrow remit simply the reversal of a single variant‐driven pathophysiology leading to disease. That PM is not always effective requires some consideration of other factors (Figure 3).

Focusing first at the single putatively pathogenic variant in a given gene, consideration of complexity could begin with all the potential consequences of that variant, across all organ systems. As detailed gene expression databases exist, and are being resolved to cellular levels, finding the culpable gene should promote and ease evaluation of such consequences. For example, knowing that the variant‐modified transcript Y of gene X is expressed in organs (or cells) A, B, and C should mean that those organs (or cells) come under scrutiny: The multi‐organ involvement in tuberous sclerosis is a longstanding example, with multidisciplinary clinics in existence in many countries; a newer example is the involvement of the heart in *ATP1A3*‐related disease.^52^

At a more intricate level, and one that is typically avoided because it is probably too complex to comprehend currently, variation across the rest of the genome, and factors (eg, the epigenome) that influence the consequences of pathogenic variation, need to be considered. There are examples of forays into such research. Accounting for the influence of additional variation within the genome beyond the putatively causal single variant may enrich genotype‐phenotype correlation and elucidate phenotypic diversity.^53^ Variants and other factors are known that influence clinically relevant features of “monogenic” conditions such as Huntington disease.^54^, ^55^ Polygenic risk scores (PRS) take into account a greater proportion of influential (common) variation across the genome and may play a numerically equivalent, or more important, role than rare variation,^56^ whereas the genome‐wide burden of rare variation itself has also been considered,^57^, ^58^ although not extensively; there are added considerations—some rare variants may be protective.^59^

Beyond data we might consider accessible and quantifiable, there is yet more complexity (Figure 3). When considering resective neurosurgical treatment for an epilepsy in an individual, best practice is to discuss relevant data in a multidisciplinary setting, weighing up not just the purely technical aspects, but also factors we may call “holistic” (for additional discussion on this important topic, see Jehi et al^60^ in this issue). Such surgical intervention falls within the broader ambit of PM—for some individuals, whether the epilepsy is considered ultimately of genetic origin or not, surgery may be the appropriate course of action (eg, for some patients with tuberous sclerosis complex or focal cortical dysplasia).^61^, ^62^ But other precision therapies may have similar effects, and should in some instances also be considered in a multidisciplinary setting. What will be the consequence of rendering an individual seizure‐free with a precision therapy? In many instances, a severe epilepsy may be associated with comorbidities such as behavioral difficulties or intellectual disability, which to an extent may improve, if some element of that disability is part of a reversible epileptic encephalopathy. What will happen if seizures stop in that setting, perhaps in a person with adult physical capabilities, but without the concomitant socialization and maturation (Figure 3D)?

Precision medicine, like epilepsy surgery, should not take place in a vacuum: It needs to consider the whole person, and it—like genomics—is part of a wider process. Although currently the vogue is primarily based on what is possible—typically drug treatment(s) based on discovery of a genetic cause in an individual—more ambitious concepts are already being realized, such as specific drugs,^63^ gene‐based therapies,^64^ antisense oligonucleotide treatment,^65^ and modulation of microRNA,^66^ as just a few examples. But PM, however, apparently targeted and sophisticated it may become, does not absolve us of responsibilities as clinicians—we have to take into account the whole person, not just the disease‐causing variant and its immediate downstream effects.

**Figure 4:**
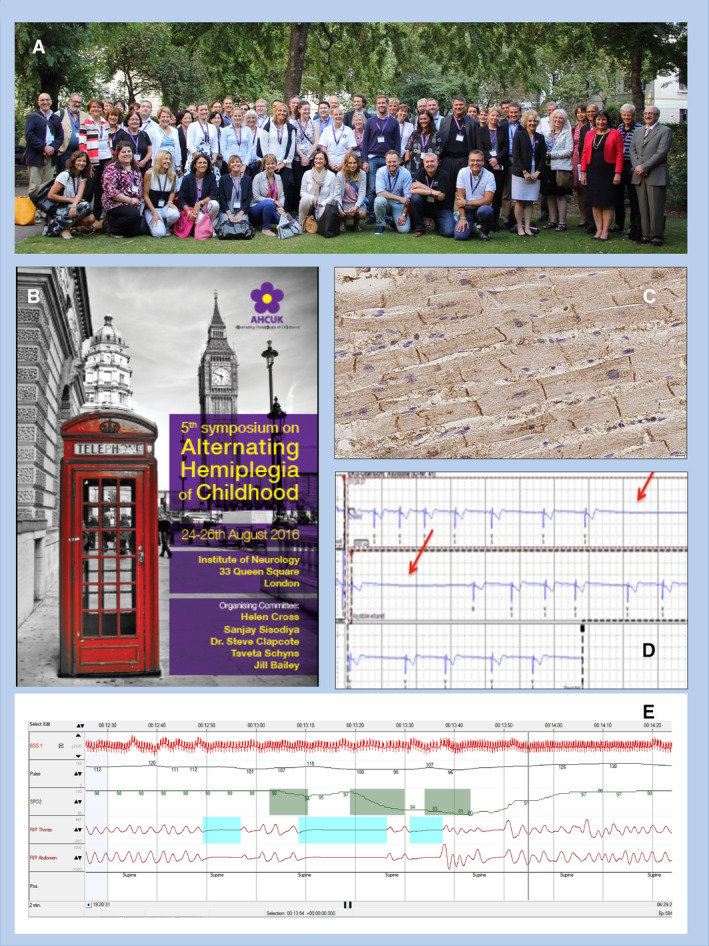
Precision medicine (PM) beyond treatment of seizures alone. The discovery of mutations in *ATP1A3* as the main cause of the ultra‐rare condition alternating hemiplegia of childhood (AHC)^74^, ^75^ catalyzed interactions between existing communities of scientists working on the protein and gene, family organizations, and clinicians and scientists interested in AHC, leading to annual joint workshops since 2012. Delegates attending the 5th Annual Symposium on AHC are featured in the photograph (A), with the meeting brochure (B). Joint working has facilitated research into important aspects of ATP1A3‐related conditions, such as cardiac involvement (C & D, respectively showing ATP1A3 immunolabeling in explanted human heart tissue and episodes of asystole in an individual with AHC)—undertaking studies in such rare conditions can be very difficult without close interaction with family organizations. Such groups can also identify and promote research questions, such as the occurrence of apnea in AHC (E), a phenomenon of deep concern to parents of children with AHC

There is potential value in adopting a more comprehensive, more complex strategy. Interaction with the individual's family, and with support groups for genetic epilepsies (or other collectives), offers the possibility of direct important insights into disease biology and disease burdens. Such exchange facilitates immediate feedback and discussion, informs longer term strategies, and moves us away from thinking about what is important for the patient to thinking about what is important to the patient and their carers. It is also typically humbling and often deeply rewarding (Figure 4).

## 5 HOW CAN WE ACCOUNT FOR AND MANAGE COMPLEXITY?

**Figure 5:**
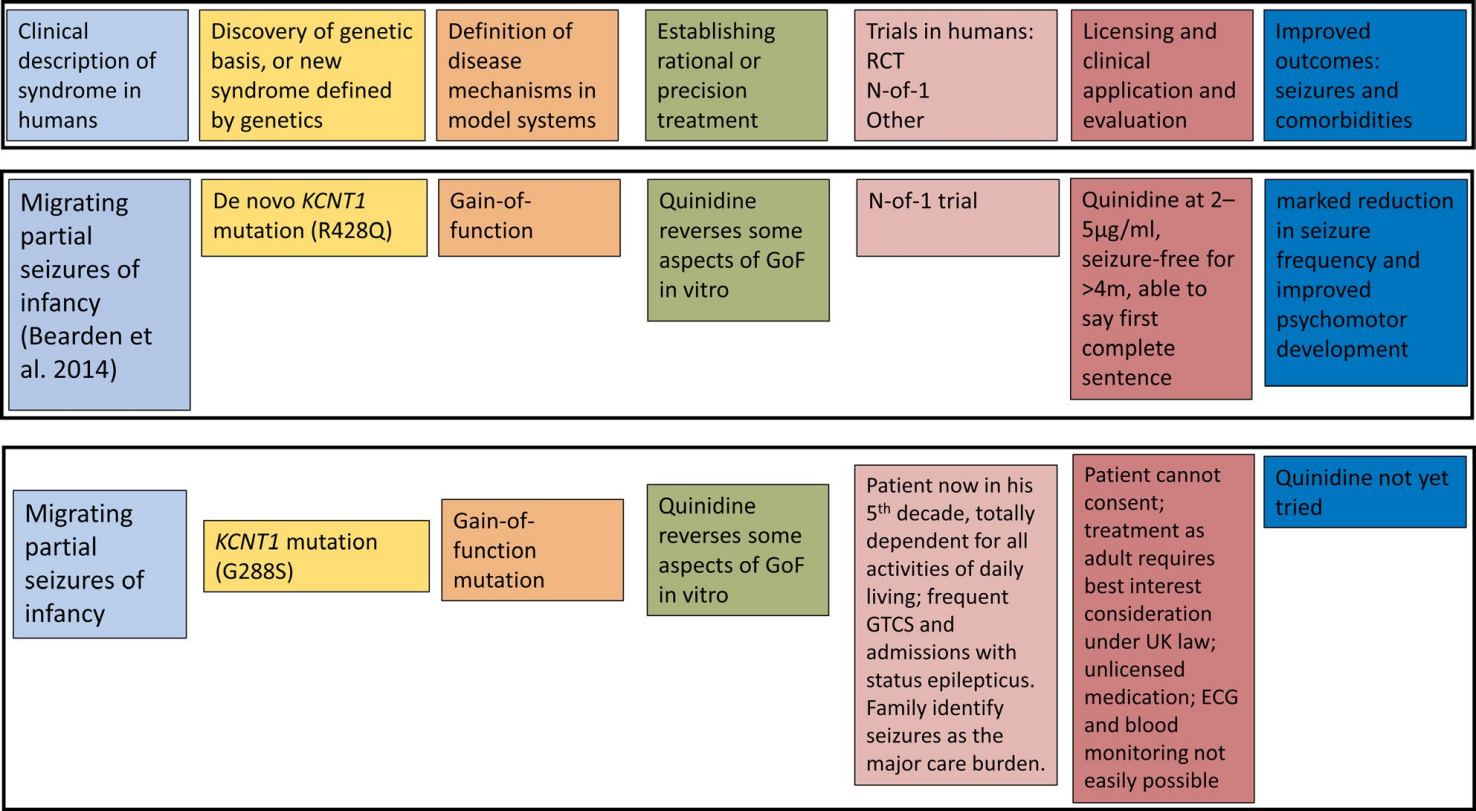
An example of difficulties in the PM approach (Top Panel) in an individual case. As documented in Table 1, quinidine was identified as a possible PM for seizures caused by gain‐of‐function mutations in *KCNT1*.^76^ Middle Panel: A child carrying a mutation studied in that first report was treated with quinidine to good effect, although the report carried information only on a short follow‐up period once seizure freedom had been obtained.^80^ Bottom Panel: A previously reported gain‐of‐function mutation in *KCNT1*, p.Gly288Ser (Ref.^78^), was detected in a man in his 40s. Parents declined testing for themselves, but based on the previous reports, the variant was considered pathogenic. Generalized tonic‐clonic seizures were considered by his parents and carers as the major burden in his life currently, repeatedly precipitating hospital admissions due to status epilepticus. However, with accumulating evidence of uncertain outcomes following quinidine treatment, including for this variant (see Table 1; Ref.^93^), concerns about cardiac toxicity, difficulties in obtaining serial electrocardiography (ECG) studies , and serum level data from the proband, who would not be able to voice symptoms himself, regulatory hurdles in the UK (which may differ from other jurisdictions), no decision has yet been made about use of quinidine in the patient

Complexity may be manageable, to some degree, when it is acknowledged and studied, and with availability of the appropriate tools. The sophistication of genomic, imaging, or electroencephalography (EEG) analyses exemplify what is possible. Analyses, for example, of cohort and trio whole exome sequence data have advanced to levels unimaginable even 5 to 10 years ago.^67^ We can be confident to high levels of certainty that a variant is real, rare, and unlikely to be found by chance in a given context, and we can put secure *P* values and confidence intervals on such estimates. We typically infer pathogenicity, although whether we can be truly confident that a variant causes a disease is perhaps sometimes less certain, especially as the search for a causal genetic variant is usually framed in terms of a monogenic pursuit. But when it comes to phenotypic characterization, we are less able. Figure 5 illustrates an example of real life difficulties in management, related to the discovery in middle age of a gain‐of‐function mutation in *KCNT1* (see also Table 1). Seizure types and syndromes are commonly defined electroclinically, but sometimes even obtaining EEG data, especially ictal data, in many people who may have genetically driven epilepsies is challenging. Securing high‐resolution MRI data may necessitate scanning under general anesthetic, which may be felt not to be justifiable. Simply defining the clinical phenomenon of multidrug resistance remains a challenge. How do we define disease severity? More fundamentally, what should even be included in “phenotype”?

At the individual level, these questions are perhaps best addressed by centering the precision medicine strategy around the affected person and their carers. Clearly, not all facets of importance in a particular epilepsy may be apparent to the affected person (for example, loss of cerebral volume at a particular instant in the disease trajectory before it is symptomatic), and these will still need to be additional points of reference. At the analytic level, ensuring inclusion of all nonredundant data sources (eg, separable aspects of genomics, imaging, and so on) may contribute to a fuller understanding. But it is also likely that we will need to devise newer means of phenotyping or include in clinical practice means of phenotyping that are currently in the research realm. Such endeavours are essential to bridge the gap between phenotype and studies of mechanisms of dysfunction using in vitro or in vivo model systems that can evaluate only a limited number of putatively causal variants in simplified systems and cannot reflect the full complexity of the phenotype in the people actually carrying the variants, a gap that itself may partly account for current failures of precision therapy strategies. Clearly, there are contrasting experiences in PM. In some cases, there is a linear solution: Standard clinical evaluation, including some form of genomics, identifies a genetic cause underlying the patient's epilepsy, with direct application of a PM, and a resulting outcome of seizure freedom. Arguably, this scenario will prove uncommon. On the other hand, things may be much more complicated. A putatively causal variant may be identified in one gene, but complexity in disease causation, drug response, outcome on treatment with a PM, and comorbidities may arise from background genomic variation, differential expression of the gene in question over time, and space within the brain, epigenomic variation, protein modification, the impact of the variant during brain development and aging, compensatory responses to variant‐driven pathophysiology and seizures themselves, and consequences of treatments with possibly inappropriate variants, with unknowable interactions between these multiple factors. Arguably, the one absolute in PM is the observed phenotype.

## 6 THERAPIES OF THE FUTURE

Excellent histories of the epilepsies document past therapies that each generation of physicians may have considered state‐of‐the‐art treatment. What will work as treatments for epilepsy in the future is a speculation. The rate of accumulation of publications in epilepsy continues to grow, and predicting which new approach will be successful is an impossible task. Questions to ask include whether there will be new drugs or therapies for disease prevention and modification, whether new strata of disease biology (eg, noncoding genetics) will provide new solutions, whether combinatorial approaches will be needed or more successful and tolerable, and indeed whether we are anywhere near defining disease mechanisms sufficiently. It seems likely that we will need to change the processes used to test new therapies, especially if these turn out to be specific to a narrow range of types of epilepsy defined by their underlying pathophysiology. In the end, PM should perhaps be considered a falsifiable hypothesis, not a linear solution. The importance of publicly documenting, in open‐access format, failures of PM on an intent‐to‐treat basis becomes increasingly clear; successes also need to clearly document the duration of follow‐up.

Precision medicine needs also to consider fairness (https://datasociety.net/output/fairness‐in‐precision‐medicine/?utm_source=STAT+Newsletters&utm_campaign=436e1528c5‐Readout&utm_medium=email&utm_term=0_8cab1d7961‐436e1528c5‐150097429): Many marginalized groups rarely feature in large‐scale studies, although newer efforts, such as Epi25, have made strenuous efforts to tackle exclusion (http://epi‐25.org/). What does and what could PM mean to the majority of people with epilepsy across the globe, who have limited access even to existing antiseizure drugs?

## 7 CONCLUSION

We need a new framework for precision medicine in the epilepsies, one that can encompass the power and concepts emerging from newer data, such as analyses of the influence of genome‐wide genetic variation. Undoubtedly any such new framework will need to be shaped by vigorous debate within the community. As a starting point for discussion, the following criteria might be considered to judge an approach as qualifying as a PM strategy:

1. There is a robust understanding of the necessary and sufficient mechanisms leading from putative cause to clinically manifest disease.
2. The postulated disease mechanisms should be measurable at the level of necessary and sufficient elements in the disease pathophysiology.
3. The precision treatment strategy should be justifiably based on the understanding of underlying mechanisms.
4. The strategy should improve clinical outcomes, with parallel evidence at the required mechanistic level that the putative pathophysiology has been corrected or addressed.
5. Failure of a precision therapy should be explained on the basis of the postulated disease mechanisms.

A precision treatment is one that meets all these criteria. It may be a set of actions, not only a single drug (for example). A precision approach embeds a precision treatment within a holistic evaluation that considers the effect of the condition and its proposed treatment on the individual as a whole in the context of their life. These criteria may be seen as over‐demanding. But if we cannot explain how our putative precision treatment is actually working and prove that it is doing what we think it is doing, then one could contest that it is indeed a precise treatment.

## CONFLICT OF INTEREST

The author has research collaborations with UCB Pharma and Congenica Ltd. We confirm that we have read the Journal's position on issues involved in ethical publication and affirm that this report is consistent with those guidelines.

## Data Availability

Requests for data are subject to data protection and ethical regulations, and they may be addressed to the corresponding author.
